# The cerebellar deep nuclei: a patch for rate codes?

**DOI:** 10.3389/fncir.2025.1548123

**Published:** 2025-04-08

**Authors:** Mike Gilbert, Anders Rasmussen

**Affiliations:** ^1^School of Psychology, College of Life and Environmental Sciences, University of Birmingham, Birmingham, United Kingdom; ^2^Department of Experimental Medical Science, Lund University, Lund, Sweden

**Keywords:** theory, deep cerebellar nuclei, network, model, code, cerebellum

## Abstract

Neural firing rates are thought to represent values which code information. There are drawbacks with using biophysical events to represent numbers. (1) Rate code (like any sequence) is inherently slow to read. (2) At short intervals, the code becomes unintelligible biophysical noise. (3) Transmission times. The vital contribution of the cerebellum to skilled execution and coordination of movements requires precision timing. We present a theory supported by modeling that the output cell group of the cerebellar network is a practical solution to timing problems. In this role, it converts irregularly-patterned firing of Purkinje cells into an effectively instantaneous rate received by output cells, transforms the rate into linear analog modulation of output cell firing, synchronizes firing between output cells, and compensates for lag caused by extracerebellar transmission times. The cerebellum is widely connected to the midbrain and the cerebral cortex and involved in cognitive functions. Modular network wiring suggests that the cerebellum may perform the same computation on input from all sources regardless of where it is from. If so, and the deep cerebellar nuclei make the same contribution to the role of the cerebellum in other functions, an understanding of motor function would also provide insight into the substrate of cognitive functions.

## 1 Introduction

The cerebellum is made up of large, anatomically overlapping cell networks. Most networks include a cell group in the deep cerebellar nuclei which includes excitatory projection neurons which carry the main output of the cerebellum (DCN, for deep cerebellar neuron). A deep nuclear group receives the output of a microzone, a functionally defined strip of cells in the outer layer of the cerebellar cortex (Oscarsson, 1979; Ozden et al., 2009; Ramirez and Stell, 2016), from Purkinje cells, which carry the sole output of the cerebellar cortex. Around 400 Purkinje cells converge onto perhaps 50 DCNs [it is likely that population sizes vary but the cell ratio is consistent with reported convergence and divergence ratios (Person and Raman, 2012b)].

DCNs fire spontaneously (Mercer et al., 2016; Raman and Bean, 1999) at robust rates, averaging about 90 Hz *in vitro* with synaptic inputs removed or blocked [interpositus in mice: 70 Hz in males, 110 Hz in females, hence the average (Mercer et al., 2016; Person and Raman, 2012a)]. A single DCN normally receives continuous torrential inhibition from 30 to 50 Purkinje cells (Person and Raman, 2012b). DCN firing rates nonetheless remain bi-directionally sensitive to a coordinated change in afferent Purkinje cell rates (Pedroarena and Schwarz, 2003). Discharge of a single Purkinje cell is irregularly-patterned (Sauerbrei et al., 2015)—the timing of single spikes is unpredictable and spike rasters have a stuttering appearance. This is true even when they are responding to perfectly sinusoidal head rotation in a vestibulo-ocular reflex paradigm (Guo et al., 2014).

The cerebellum is critical for skilled execution and fine coordination of movements, requiring precise timing. Neuron firing rates are thought to code values that are processed in neural network computations. There are drawbacks with using biophysical events to represent numbers. (1) Rate code (like any sequence) is inherently slow to read. (2) At short intervals, the code becomes unreadable biophysical noise. (3) Neuronal and synaptic transmission times cause lag between the time of events that generate input to the cerebellum and the time that motor outputs are received in response. Both send and read times impose significant delays, on the face of it too long for cerebellar coordination of motor control at behavioral speeds.

We propose that the cerebellum solves the slow read time problems (1 and 2) by coding information at the scale of cell groups which communicate at that level, group to group. From that premise, we can explain a range of evidence that has previously been hard to account for (or thought of as marginal to analysis), including anatomical architecture, cell morphologies, firing properties (different for different cell types) and specializations of synaptic contact. There is, we propose, a passive but fundamentally important computational effect of anatomy. This is the result of a combination of elements. The main elements are:

(a) Strong convergence of Purkinje cells onto DCNs (because Purkinje cells outnumber DCNs and also make divergent contact, increasing the convergence ratio), so that a single DCN receives a very high combined rate of spike input even at low individual afferent rates.
(b) Contact by a single afferent cell via multiple boutons which are scattered across the DCN soma surface and intermingled seemingly at random with the boutons of other afferent cells, so that charge transfer into the postsynaptic cell is directly into the DCN soma, at all locations of the soma surface, at a rate which is the same everywhere.
(c) Each afferent spike causes a standard unit of inhibitory charge entry notwithstanding the normally expected mutual effect of membrane voltage and current. As a result, the sum of charge inflow/time linearly reflects the mean of afferent rates in very short time intervals—effectively, an instantaneous rate.

Anatomy also coordinates network output. Contact on a single DCN is made by a—seemingly, and we suggest—random sample of Purkinje cells afferent to the DCN group. Effectively, DCNs randomly sample Purkinje cell rates. As a result, DCNs individually receive, at any instant in time, a mean rate of input which is close to the mean for the sampled population (the population of Purkinje cells). Accordingly, they receive close to the same mean rate as each other, so that firing of DCN rates in an output group is synchronized.

We propose that the cerebellum solves problem 3 by exploiting short-term plasticity at the Purkinje cell-DCN synapse. Firing of Purkinje cells is ceaseless but at a time-varying rate. Synaptic transmission is simultaneously depressed (Pedroarena and Schwarz, 2003; Telgkamp and Raman, 2002) and enhanced (Turecek et al., 2016, 2017) by independent synaptic mechanisms with a mutually antagonistic effect. On the face of it, this is a puzzle. At a constant afferent Purkinje cell firing rate, the effects net off. However, during behavior, when the Purkinje cell rate is changing, the net effect—we propose—depends on the direction and rate of change of the Purkinje cell rate. The result is that in a locomotor cycle, the modulation of DCN firing rates in response to Purkinje cell control is phase shifted to an earlier time, potentially compensating for lag caused by signal transmission times, including extracerebellar times. The amount of time—the absolute shift—is adjusted for wavelength, so that compensation is by a fixed adjustment regardless of wavelength.

It will be assumed that the time-varying probability of a Purkinje cell action potential is synchronized between Purkinje cells in a microzone. It has been proposed previously how this may be coordinated (Gilbert and Rasmussen, 2025). For the argument we make in this paper, it is unnecessary that Purkinje cell firing is coordinated in the manner proposed previously, only that it is coordinated.

The deep cerebellar nuclei have other outputs (Kebschull et al., 2024). For example, there are projections to the inferior olive and back to the cerebellar cortex. Our focus is the conversion of Purkinje cell firing into control of DCNs. Boxes are used for presentation of related ideas that accompany the main narrative. References to Purkinje cell spikes are to simple spikes.

## 2 Purkinje cell rate information is collectively coded in a time-varying probability of simple spike discharge

Irregular spike timing is a common phenomenon in the brain, even under tight experimental control (de Ruyter, van Steveninck et al., 1997; Mainen and Sejnowski, 1995; Schreiber et al., 2004). Purkinje cell somata form a thin single-cell layer, the Purkinje cell layer, which lies between the outer and inner layers of the cerebellar cortex and parallel to the cerebellar surface. Irregular firing of Purkinje cells appears to be caused at least partly by stellate cells, interneurons in the outer layer of the cerebellar cortex which inhibit Purkinje cells. Oscillations of field potential in the Purkinje cell layer can cause spike timing to become synchronized (Han et al., 2018). Irregular Purkinje cell spike timing may be actively induced to prevent firing from falling into an oscillating pattern (Box 1).

**BOX 1 Irregular spike timing of Purkinje cells is induced so that control of DCNs is by rate information only, without interference from spike timing (we submit).**

Stellate cells form planar networks (Palay and Chan-Palay, 1974; Sultan and Bower, 1998), making and receiving synaptic contact at the soma (Lemkey-Johnston and Larramendi, 1968). Stellate cells themselves fire irregularly *in vitro* (Carter and Regehr, 2002; Häusser and Clark, 1997; Ruigrok et al., 2011) and *in vivo* (Barmack and Yakhnitsa, 2008; Jörntell and Ekerot, 2002), probably the result of inhibiting each other, as they fall into a regular firing pattern when inhibition is blocked (Häusser and Clark, 1997). Inhibition of Purkinje cells by stellate cells causes regular intrinsic firing of Purkinje cells to become irregular (Häusser and Clark, 1997; Jelitai et al., 2016), and selective silencing of molecular layer (the outer layer of the cerebellar cortex) interneurons causes Purkinje cell firing to fall back into a more regular pattern, and leads to motor deficits (Jelitai et al., 2016; Wulff et al., 2009). Even a single stellate cell spike can potently inhibit a Purkinje cell (Arlt and Häusser, 2020).

Purkinje cells generate ephaptically coupled field potentials in the Purkinje cell layer which promote spike synchrony (Han et al., 2018) in experimental conditions. As a result, left to themselves, neighboring Purkinje cells fall into an oscillating firing pattern. Purkinje cell-mediated inhibitory postsynaptic currents decay quickly with a constant of ∼2.5 ms (Mercer et al., 2016; Person and Raman, 2012a). Even brief gaps between IPSCs make inhibition-reduced windows in which DCN spiking is more likely (Wu and Raman, 2017). This is seen in the first couple of rounds of spikes after stimulation of the mouse whisker pad, when spikes are briefly synchronized (Brown and Raman, 2018).

Such an effect of oscillations on DCN firing is an effect of spike timing. If this were to occur in natural conditions, it would interfere with control exclusively by afferent rates. Interference would be noise if—as we propose—information is coded only in rates and not in spike timing. Irregularizing Purkinje cell firing may be a strategy to disrupt ephaptic coupling, with the function of noise reduction by eliminating an influence on DCN firing of oscillating field potentials. Purkinje cell recurrent collaterals (Witter et al., 2016) may share this function with stellate cells.

We hypothesized that Purkinje cell rate information is coded in the moment-to-moment probability of simple spike discharge. At any moment, in any cell, the outcome is binary (spike or no spike), and independent of other cells. However, the probability is synchronized between Purkinje cells in a microzone.

This proposition is consistent with the appearance of spike rasters in behaving animals. In free-to-move rats, the spiking pattern of a single cell is unpredictably variable but gives a smooth curve when spikes are counted in phase-locked bins across multiple step cycles (Sauerbrei et al., 2015). Extracellular recordings from Purkinje cells show a phase-dependent increase and decrease in their firing rate during locomotion (in cats: Armstrong and Edgley, 1984, 1988; Edgley and Lidierth, 1988).

**FIGURE 1:**
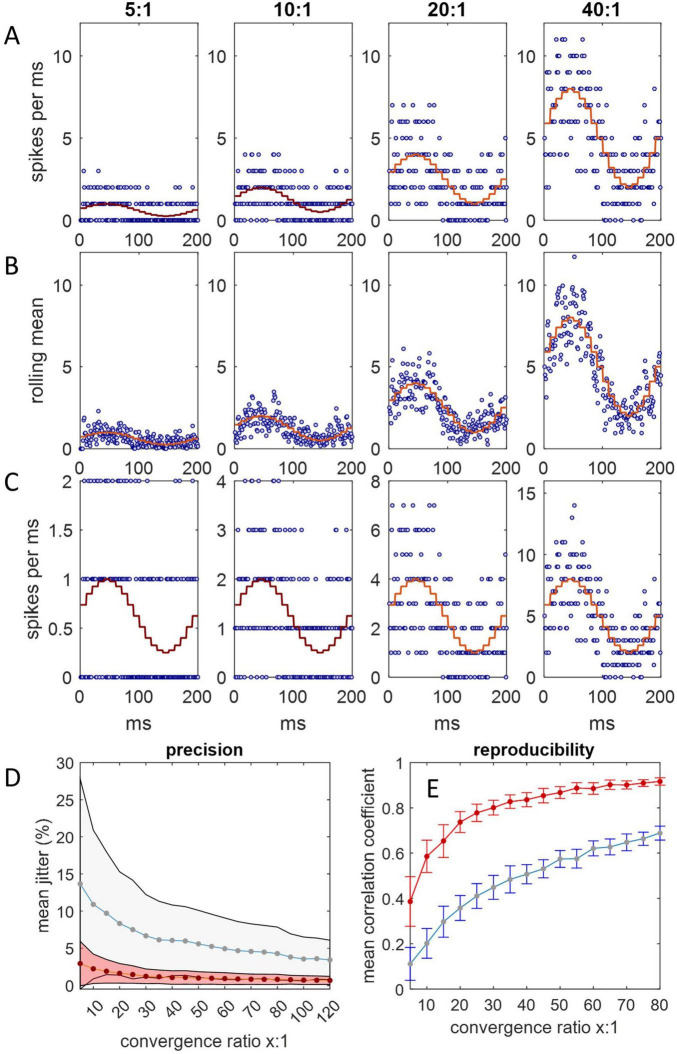
Spike input to a single DCN. Simulations were run in Matlab. **(A)** Spike input to a single DCN from 5, 10, 20, and 40 afferent Purkinje cells, each millisecond, for 200 ms, representing a step cycle (of a rat: Sauerbrei et al., 2015). Spikes are generated with a sinusoidally varying probability representing an instantaneous firing rate oscillating between 50 Hz and 250 Hz, and counted in millisecond bins. At each timestep, the probability is the same for all Purkinje cells, but the outcome (1 or 0 spikes) is independent. The stepped red line is the expected firing rate at each probability. Convergence ratios are shown above each column. **(B)** We took a rolling mean of consecutive counts as a proxy of postsynaptic integration. The combined spike input rate to a DCN is well predicted by the probability function. **(C)** The same as (A) but y axes are scaled to the data to illustrate noise reduction relative to the data range. The coefficient of variation is inversely related to the convergence ratio. **(D)** The relationship of relative precision and the convergence ratio. Jitter is defined as the difference in the spike count between consecutive 1 ms bins, normalized for the convergence ratio (by dividing by the number of afferent Purkinje cells), averaged across a 200 ms step cycle. Plotted data are mean jitter (+/– 1 SD) across 20 cycles with integration (red data) and without (blue), expressed as a percentage of the number of afferent Purkinje cells. **(E)** The mean correlation coefficient (+/– 1 SD) of all possible pairs in 20 simulated datasets of a 200 ms step cycle, plotted for each convergence ratio in the range *x* = 5:1 to 80:1. Most of the increase in reliability is in the lower end of the range, with diminishing returns at higher ratios.

We reasoned that if Purkinje cells afferent to a DCN group spike with a synchronized time-varying probability, the combined input spike rate to a DCN in a single cycle should have the same appearance as reported recordings from a single Purkinje cell across multiple cycles. To replicate the physiological spiking pattern of Purkinje cells, we represented Purkinje cells as random spike generators that discharge with a changing probability that oscillates sinusoidally between minimum and maximum instantaneous firing rates of 50 Hz–200 Hz, mimicking the rat (Sauerbrei et al., 2015). DCNs were represented by counting spikes in millisecond bins for 200 ms, with Purkinje cell-to-DCN convergence of 5:1, 10:1, 20:1 and 40:1 (40:1 is the physiological mean), taking a rolling mean of consecutive counts as a proxy for postsynaptic integration. We found (Figure 1) that the integrated combined rate of spike input to a DCN reliably reflects the underlying sinusoidal probability function: a synchronized probability of Purkinje cell spike discharge is plausibly able to code rate information (with 40:1 convergence). Noise reduction and sensitivity are enhanced by increasing the convergence ratio (Figures 1B, C). The rate of increase of noise reduction diminishes as the convergence ratio increases (Figure 1D). The effect on noise reduction of an increase in convergence that exceeds the physiological ratio is relatively modest. There is little gain in reproducibility by increasing convergence above the physiological range (Figure 1E).

## 3 A single spike drives inflow to the DCN soma of a standard unit of inhibitory charge per spike per bouton

Purkinje cells make contact on DCNs at the soma, via boutons (de Zeeuw and Berrebi, 1996; Uusisaari and De Schutter, 2011). A bouton encloses the space that GABA is released into. A DCN receives 24–36 boutons from each Purkinje cell (Person and Raman, 2012b; Telgkamp et al., 2004). A single DCN receives significant contact from 30 to 50 Purkinje cells, so that the DCN soma surface is engulfed in Purkinje cell boutons. The modulation of inhibitory postsynaptic current is bidirectional, sensitive and fast (Pugh and Raman, 2005; Telgkamp et al., 2004).

It forms part of our proposal that each input spike to a single bouton causes a standard unit of charge transfer across the postsynaptic membrane (“charge” in this paper refers to inhibitory charge unless qualified). This provides the core element of a linear relationship of Purkinje cell discharge probability and the rate of charge movement across the postsynaptic membrane. Perineuronal nets—an extracellular matrix that surrounds the DCN soma—may assist the fidelity of this relationship, as they reduce paired-pulse depression and spontaneous inhibitory postsynaptic currents (Hirono et al., 2018). As in this proposal charge inflow per spike is invariant, charge/time is a linear function of total spikes/time.

A linear relationship feels counterintuitive because it does not allow for the expected mutual effect of transmembrane voltage and current. However:

(i) A linear relationship has been reported—the charge transfer rate is linearly related to a constant Purkinje cell firing rate (Turecek et al., 2016, 2017). In mouse slices, it has been reported that “the Purkinje cell-DCN synapse differs from many other synapses in that frequency-independent transmission leads to linear charge transfer [at a constant Purkinje cell firing rate], which could encode the absolute rate of Purkinje cell firing more efficiently than a typical depressing synapse” (Turecek et al., 2016, 2017); and
(ii) Purkinje cell boutons are adapted to isolate the relationship by controlling for other variables (Box 2).

**BOX 2 Physiological adaptations of the Purkinje cell bouton reduce noise.**

Variables that affect transmission but code nothing are noise. Purkinje cell-DCN contact is adapted to isolate the relationship of Purkinje cell discharge probability and charge transfer by controlling for other variables, we submit. There are presynaptic and postsynaptic adaptations.Presynaptic: Presynaptically, boutons contain a large number of neurotransmitter release sites. Individually low vesicular release probability by a large number of presynaptic vesicles (Pugh and Raman, 2009) reduces spike-to-spike variability of release. Reuptake by astrocytic transporters is confined to the bouton perimeter so that rapid GABA diffusion inside a bouton ensures that postsynaptic densities are activated as a single functional unit.

Intrabouton summation of consecutive releases is absent provided the interspike interval at physiological Purkinje cell rates is not shorter than the time needed for intrabouton GABA concentration to be restored to baseline. At 200 Hz, the time available is ∼5 ms. For comparison, the decay constant of inhibitory postsynaptic current is ∼2.5 ms (Mercer et al., 2016; Person and Raman, 2012a).

Cross-integration between boutons is absent because boutons are physically bounded. Otherwise, the surface of the DCN soma would be bathed in a GABA cloud.

Postsynaptic: Charge entry to the DCN soma evoked by a single afferent Purkinje cell—indeed a single spike—is delivered at multiple boutons [24–36 in mice (Person and Raman, 2012b)] that are dispersed across the soma surface. These are intermingled with boutons of other Purkinje cells. As far as we know, boutons are randomly sited. Some 1,200 boutons, provided by an average of 40 Purkinje cells, cover the somatic surface. The combined input spike rate (from ∼40 Purkinje cells, each firing continuously at typically robust rates) is therefore very high. The functional effect is of convergence onto a point, i.e., while present, spatial and temporal integration are not variables. So, noisy variables associated with discrete signals, and variables associated with receiving signals dendritically, are absent.

**FIGURE 2:**
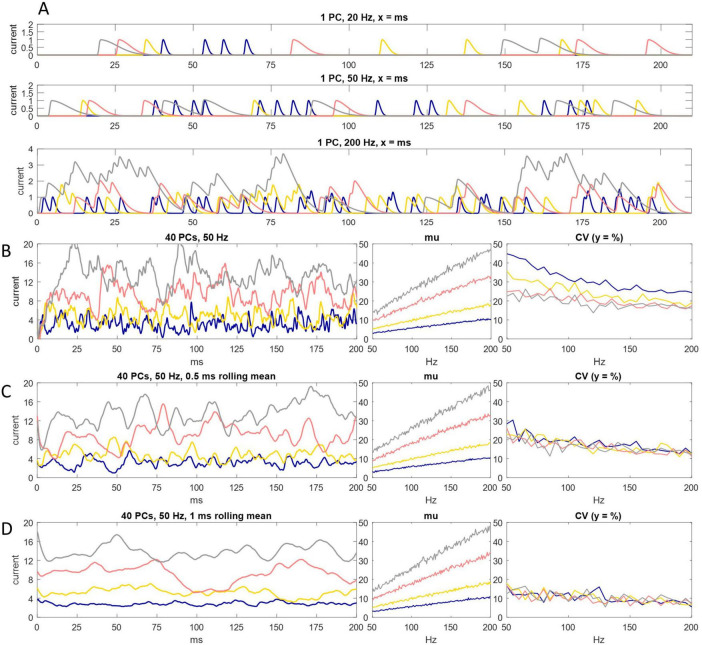
Charge entry to the DCN soma. **(A)** Simulation of inhibitory charge entry to the DCN soma driven by a single Purkinje cell firing at an instantaneous rate of 20 Hz (top panel), 50 Hz (middle panel) and 200 Hz (bottom panel), for 200 ms. A Purkinje cell is represented as a random spike generator. Spikes are generated with a probability of N/20,000, in each timestep (of 0.05 ms), with a refractory period of 1 ms, where N is the instantaneous rate. Each spike triggers a standard quantum of simulated charge entry to the postsynaptic cell. The quantum depends on the decay constant of inhibitory current. Current has a decay constant of 1.25 ms (blue), 2.5 ms (yellow), 5 ms (pink) and 7.5 ms (gray). All signatures have the same rise time and peak amplitude. Peak amplitude of a unit of charge entry is take as a unit of current equal to 1. Where quanta of the same color overlap in time, current sums linearly. Even at 200 Hz summation is intermittent. It is sparse with a short decay constant. **(B)** Left: the same as **(A)** but input is from 40 Purkinje cells, firing at an instantaneous rate of 50 Hz. Current summates reliably but not smoothly. Middle and right: we ran the simulation 200 times, increasing the Purkinje cell discharge probability by the equivalent of 1 Hz each time, from 50 Hz to 200 Hz, and each time calculated the mean of current data and coefficient of variation (CV) in each condition (i.e., for each decay constant). There is a linear relationship between Purkinje cell discharge probability and the mean rate of charge entry to the DCN soma in all conditions. **(C,D)** We took a moving average of current data in a rolling time interval of 0.5 ms **(C)** and 1 ms **(D)**. In conditions that in **(B)** cause random fluctuation of current data at high frequency and low amplitude (i.e., with a short decay constant), data converge toward the 200 ms mean, so that they approach a linear relationship with Purkinje cell discharge probability. That is, smoothing the data also increases precision. With a long decay constant, data converge weakly toward the mean (data are smoothed but there is little increase of precision).

We simulated spike input to the DCN soma to test if charge transfer is proportional to the probability of Purkinje cell spike discharge (Figure 2). We found that fidelity of the relationship depends on the decay constant of postsynaptic current.

We represented Purkinje cells as random spike generators that discharge with a probability derived from firing rates in the rat physiological range during locomotion, as before. Each spike initiates an episode of charge entry to the postsynaptic soma (Figure 2A) with a fast rise time and a decay constant which we varied. A single unit of current is taken as peak amplitude of charge entry caused by a single input spike. Temporally overlapping episodes summate. Current was summed in intervals of 0.05 ms. Summation of current is linear—the quantum and dynamics of a unit of charge entry are inviolable.

In all tested conditions—i.e., regardless of the duration of the decay constant—the mean of current data taken over 200 ms precisely encodes Purkinje cell discharge probability. However, individual current data vary around the mean at shorter time intervals. Unexpectedly, variation is not reduced by increasing the length of the decay constant. Rather, while data vary more smoothly, they vary in a larger range, further from the mean. A short decay constant is more precise (current data are more tightly grouped) but also more jittery (inside that range, current fluctuates randomly at high frequency).

## 4 A sub-millisecond rolling mean dampens jitter of current data

We reasoned that fast but not instantaneous voltage-proportional charge expulsion from the DCN soma may mimic a rolling mean with a short decay constant. Our reasoning is that over an unknown minimum time interval, the amount of charge that crosses the DCN somatic membrane in both directions nets off. Otherwise, there would be a progressive accumulation or depletion of negative charge inside the postsynaptic cell. We envisage that removal of negative charge is fast, at a rate which varies proportionally with negative membrane potential, but not instantaneous. Because there must be at least some lag, the rate of removal cannot precisely keep pace with the rate of entry, so there must be (short-term) dampening of the rate of change of membrane potential, to preserve net-off. We mimicked that by averaging current in short time intervals.

Taking a rolling mean of postsynaptic current data even in a very short rolling time interval profoundly dampens jitter. With a short (but not a long) decay constant, dampening causes current data to converge strongly toward a straight line at a constant afferent Purkinje cell firing “rate” (i.e., unchanging spike probability).

We took a rolling mean with different decay constants (Figures 2C, D). We saw previously that a short decay constant causes inward current to fluctuate at high frequency and low amplitude. In those conditions, a rolling mean, even taken at very short time intervals (sub-millisecond), causes the data to converge strongly toward the 200 ms mean at a constant Purkinje cell discharge probability. That is, smoothing the data has the result of also increasing precision. This effect diminishes strongly with a longer decay constant (smoothing does not increase precision, or does so less).

We used a very short rolling interval to reflect a prediction that charge expulsion from the soma is fast. Functionally, fast expulsion means that somatic charge closely reflects the very recent rate of charge entry, and therefore recent information.

**FIGURE 3:**
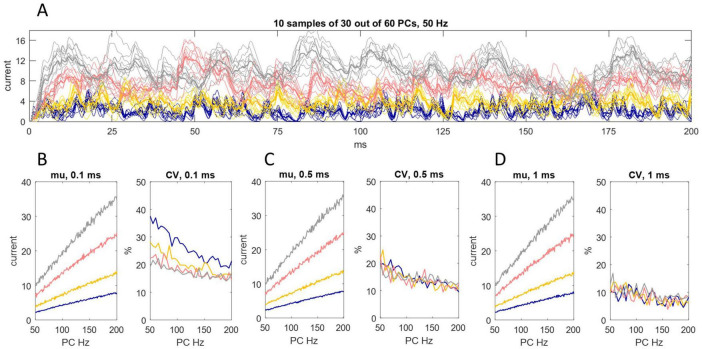
Random sampling of Purkinje cells by different somatic sites. **(A)** We simulated the division of the DCN somatic surface into 40 subregions, each receiving contact from a random sample of 30 out of 60 Purkinje cells afferent to the cell. That generated 40 data sets of 4,000 values each, representing the rate of charge entry to each site each ms/20 for 200 ms. We took the mean of data at each time point to represent the rate of charge entry to the cell. The results are shown for each decay constant using the same color code as earlier Figures. Sampling is out of 60 because the total number of Purkinje cells afferent to a DCN is higher than the estimate for “significant” contact. Thin lines are the rate of inhibitory charge entry to 10 out of 40 sites (selected at random; only 10 are shown to thin the data for presentation). Bold lines are the rate of charge entry averaged across all locations. **(B–D)** We took a 0.1 ms **(B)**, 0.5 ms **(C)** and 1 ms **(D)** rolling average of data generated in this way, for Purkinje cell discharge probabilities equating to firing rates in the range 50 Hz–200 Hz, and calculated the mean and coefficient of variation (CV) in each condition (i.e., each permutation of rolling interval, decay constant and discharge probability). The linear relationship of Purkinje cell discharge probability and the 200 ms mean of current data is unaffected. There is a slight reduction of the range of oscillation around the mean but the effect is modest.

We next tested whether regional differences of input to different sites of the DCN somatic surface make a difference to the effect received by the postsynaptic cell. We simulated the division of the DCN somatic surface into 40 subregions (Figure 3). The linear relationship of Purkinje cell discharge probability and the 200 ms mean of inward current is unaffected. There is a slight contraction of the range of current data but not enough to infer physiological significance from *in silico* results.

## 5 DCNs in a nuclear group fire at a synchronized rate

**FIGURE 4:**
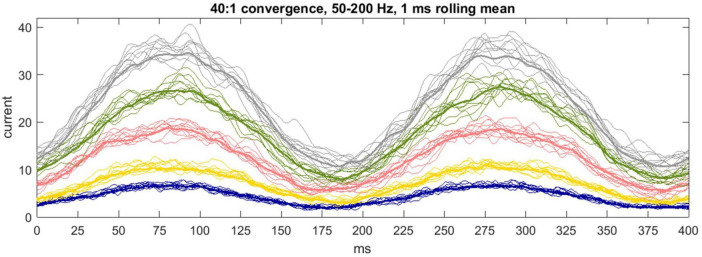
Communication by a microzone with a nuclear group. 1 ms rolling average of inhibitory current passing into the soma of a DCN each 1/20 of a ms for 400 ms. The color code is the same as earlier figures. Input to a cell is from 40 out of 400 Purkinje cells afferent to the nuclear group (40:1 is the mean physiological convergence ratio), each firing with a sinusoidally fluctuating probability of spike discharge mimicking firing across the rat step cycle. The simulation generates 50 data sets per color, representing a nuclear group of 50 DCNs, of which a randomly selected 10 are displayed to declutter presentation of the data. The bold line is the mean. Precision is inversely related to the duration of the decay constant, as it was at a constant Purkinje cell “rate.” The reported decay constant for mice is between the yellow and pink data. These are hypothetical conditions—in physiological conditions current receives an influence of short-term plasticity (Figure 6).

There is no reported internal organization of the pathway from a microzone to a DCN group (Apps et al., 2018 p. 663), or of the way that Purkinje cells terminate. On the face of it, and we propose, a single DCN receives contact from a random sample of Purkinje cells afferent to a DCN group. Anatomy simulates sampling with “replacement”—there is no influence of the make-up of the sample received by a DCN on the make-up of the sample received by any other DCN. Accordingly, DCNs independently randomly sample Purkinje cell firing rates. This is functional. The sum (or mean) of independent random samples has a frequency distribution with a narrower range than the sampled distribution, so random sampling causes the postsynaptic effect to contract toward synchronized modulation of DCN somatic charge (Figure 4).

Physiological performance is unlikely to produce exact co-modulation of DCN firing. Decay constant durations are not exactly equal, boutons are not exactly standard, and the number of Purkinje cells afferent to a DCN, and boutons received from each Purkinje cell, are random variables. We explore in Box 3 a mechanism which may tighten synchrony. Note: In physiological conditions, inhibition of DCNs receives modulation by short-term plasticity, discussed in section “6 Short-term plasticity may compensate for lag caused by transmission times.” Figure 4 is a hypothetical case—the simulation does not include short-term plasticity—to illustrate that biological exploitation of statistics has reliable computational output.

**BOX 3 DCN axon collaterals may tighten synchrony of DCN firing in a nuclear group.**

**FIGURE 5:**
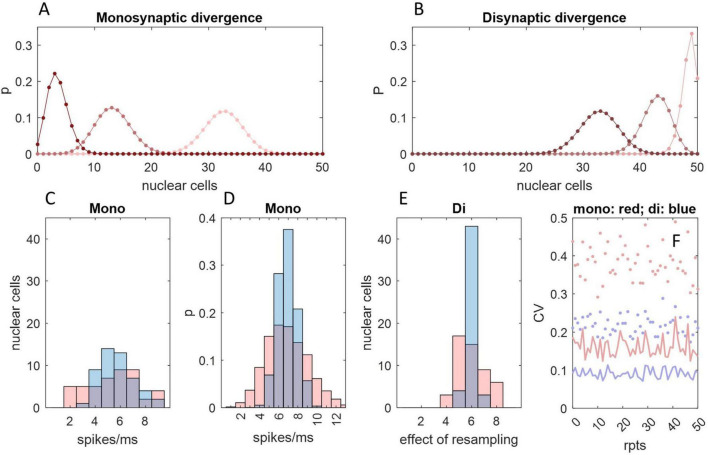
An interneuronal effect of DCN collaterals. **(A)** The probability that a number of DCNs, *x*, all receive contact from 1 or more (pink), 2 or more (red), or 3 or more (dark red) out of a random sample of 10 Purkinje cells, which each make contact at random on 5 DCNs. The number of Purkinje cells is chosen for illustration. The probability distribution was derived from the probability that a given DCN (out of a group of *n* = 50) receives contact from *y* out of *m* Purkinje cells, given by P⁢(y)=(zn)y*(1-(zn))m-y*(my) with Purkinje cell to DCN divergence of 1:*z*, so that the probability that a given number of DCNs, *x*, receives contact from at least *y* out of *m* Purkinje cells is P⁢(y⁢o⁢r⁢m⁢o⁢r⁢e)=[1-∑j=0y-1P⁢(y)]x*[∑j=0y-1P⁢(y)]n-x*(nx). **(B)** The same as **(A)** but representing nucleo-nuclear contact made by recurrent axon collaterals of a subset of 33 DCNs—the average number that receive contact from at least 1 Purkinje cell in **(A)**—again with divergence of 1:5 and assuming contact is at random within a nuclear group. Just 10 Purkinje cells—only 2.5% of the population afferent to a nuclear group—exert a secondary effect on all or almost all DCNs, and an average of 86% DCNs receive convergent input i.e., excitation from 2 or more of their neighbors. **(C)** Frequency distribution representing one millisecond of a step cycle showing the number of Purkinje cell spikes received by each DCN in a nuclear group, with (blue) and without (pink) taking a rolling mean, with a Purkinje cell discharge probability equivalent to a firing rate of 147.5 Hz (the fifth timestep in the Figure 1 cycle, selected at random), assuming a DCN population of 50 and Purkinje cell to DCN convergence of 40:1. **(D)** The frequency distribution in **(C)** reflects a probability distribution (same color code). **(E)** DCNs give rise to axon collaterals which are believed to terminate back in the same group. If so, and collaterals terminate at random inside a group, a DCN effectively randomly samples firing rates of its neighbors. The distribution in panel **(C)** was randomly sampled 50 times (representing 50 DCNs), sample size 5 (reflecting an assumption that divergence is 1:5). Sample means were plotted as a frequency distribution. Resampling narrows the distribution. The *x*-axis label alludes to the fact that the data are not a spike count *per se*. **(F)** Dots: we repeated **(C)** 50 times, calculating the coefficient of variation (using the same color code). Lines: the same but repeating **(E)**. Focus is tightened by resampling.

DCN recurrent collaterals may increase disynaptic convergence of Purkinje cells onto DCNs, causing DCN firing rates to be more tightly grouped. The deep cerebellar nuclei contain what were at one time presumed to be glutamatergic interneurons (Uusisaari and Knopfel, 2008) which are intrinsically active but normally held under inhibitory restraint by Purkinje cells. Therefore, a fall in Purkinje cell rates, which disinhibits nuclear projection neurons, also causes interneurons to excite them, and vice versa. More likely, presumed interneurons are in fact collateral axon branches of DCNs (Kebschull et al., 2020). If axon collaterals terminate back in the same nuclear group, so that a group provides excitatory input to itself, it would substantially increase functional divergence of Purkinje cells onto DCNs, i.e., direct contact plus disynaptic influence (Figure 5). Even a very small fraction of Purkinje cells (2.5%) afferent to a nuclear group is sufficient to influence the whole or almost the whole of a DCN group disynaptically. For the same reason, there is a high disynaptic convergence ratio—a large fraction of Purkinje cells afferent to a nuclear group have either a direct or disynaptic influence on a single DCN.

Figure 5C shows the frequency distribution of the number of Purkinje cell spikes received by each DCN in a single timestep (1 ms bin) of the cycle in Figure 1, with and without taking a rolling mean. Each DCN receives input from a random sample of 40 Purkinje cells (the physiological mean) out of 400 afferent to a simulated nuclear group of 50 cells. We then resampled the Figure 5C distribution (50 times, representing 50 DCNs), to simulate innervation by DCN axon collaterals. We set the nucleo-nuclear divergence ratio at 1:5 (it is unknown). Therefore, sample size was five, the mean convergence ratio. Randomly sampling a normal distribution gives another, narrower distribution, seen in Figure 5E. The result is a tightly-focused distribution even at an extremely short time scale. Excitatory synaptic contact on DCNs is primarily (75%) dendritic (de Zeeuw and Berrebi, 1996), plausibly providing tonic excitatory smoothing of the effect of direct inhibition.

## 6 Short-term plasticity may compensate for lag caused by transmission times

The cerebellum is reciprocally connected with the cerebral cortex (Strick et al., 2009), and is involved in sensory-motor loops. This section argues that Purkinje cell-DCN short-term synaptic plasticity may provide a mechanism that compensates for extracerebellar transmission times.

Purkinje cell to DCN transmission simultaneously receives opposing effects of short-term changes which both depress (Pedroarena and Schwarz, 2003; Telgkamp and Raman, 2002) and facilitate (Turecek et al., 2016) inhibition of the postsynaptic cell. In a steady state—i.e., when Purkinje cells maintain a constant firing rate—there is a linear relationship of Purkinje cell firing and charge transfer, so that inhibition scales with the absolute rate (Turecek et al., 2016, 2017). However, depression is slower to adapt to a change in the Purkinje cell rate. As a result, when the rate changes, there is a delay (of around 100 ms: Telgkamp and Raman, 2002) before the steady-state balance between depression and facilitation is restored. This makes the nuclear response sensitive to change of the afferent rate, rather than (only) the rate itself.

During locomotion, Purkinje cell firing rates change ceaselessly, passing through a repeating cycle; some cycles resemble a sine wave (Edgley and Lidierth, 1988; Sauerbrei et al., 2015). Cycle duration is sub-second—200–300 ms in the mouse (Sarnaik and Raman, 2018). Accordingly, there is a dynamic state of imbalance of short-term depression and facilitation. As a result, during behavior, the DCN firing rate receives an influence of the rate of change of the Purkinje cell rate as well as the rate itself.

We hypothesized that the net effect depends on the direction and speed of change of the Purkinje cell firing rate. When the Purkinje cell firing rate falls, lag means that short-term depression is stronger than it would be at the same firing rate if the rate was constant. As a result, there is net short-term depression that is proportional to the rate of fall, and therefore weaker inhibition than steady-state inhibition at the same firing rate. When there is a rising Purkinje cell firing rate, this is reversed, that is, there is net facilitation (again proportional to the rate of increase), and therefore stronger inhibition than steady-state inhibition at the same firing rate. A faster rate of change in either direction causes transiently greater inequality between depression and facilitation, and therefore a larger effect.

**FIGURE 6:**
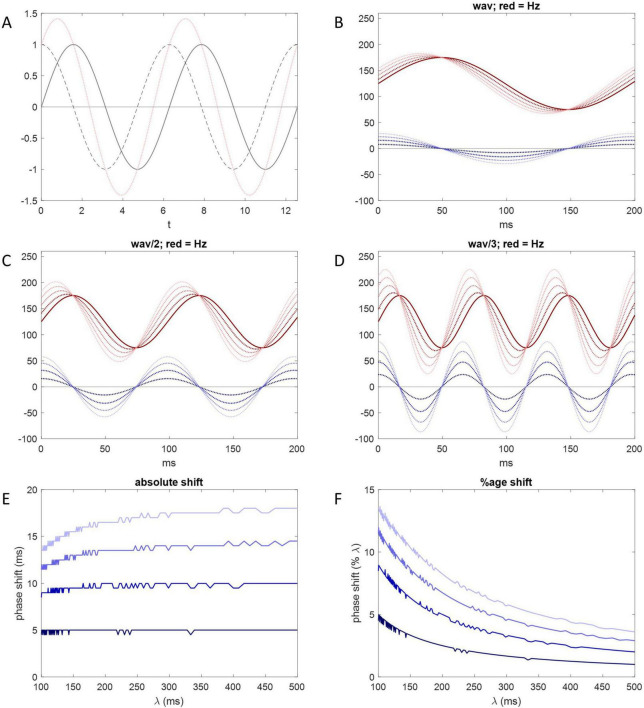
Short-term plasticity may phase shift Purkinje cell inhibition of DCNs. **(A)** Solid gray line: *y* = sin(*x*); dotted gray line: $\frac{d\,y}{d\,x}$, the derivative; pink line: the sum of the solid gray line and dotted gray line data—there is a 90-degree phase shift of the derivative and a 45-degree phase shift of the sum. **(B)** Solid red line: $y=a\,\left(\sin\left(z\,\frac{x}{\pi }\right)\right)+b$, where (for illustration) *a* = 50, the size of the range of the sinusoidally-varying Purkinje cell firing rate either side of *b*, and *b* = 75. *x* represents time and *z* is used to adjust wavelength. The solid red line represents membrane voltage of the DCN soma across a locomotion cycle under the sole influence of the Purkinje cell rate, without an adjustment by short-term plasticity. Dark blue dotted line: $\frac{\partial f}{\partial x}$, the derivative of the red function in respect of *x* keeping other variables constant. Blue amplitude represents the size of the effect of short-term plasticity. Large amplitude represents more relative influence (in a balance with influence of the absolute Purkinje cell rate). Dotted red lines: the sum at each time point of the solid red line and one of the dotted blue lines. Dark to light blue corresponds to dark to light red. For example, the darkest red dotted line is the sum of the solid red line and the darkest blue dotted line. The result in all cases is a phase shift to the left. Increasing blue amplitude increases the size of the phase shift. **(C,D)** Same as **(B)** but wavelength is halved in **(C)** and further reduced in **(D)** (by changing *z*). Shortening red wavelength increases blue amplitude because blue amplitude varies with the rate of change of the Purkinje cell firing rate. Accordingly, shortening red wavelength increases the size of the phase shift measured in degrees, i.e., relative to wavelength. **(E)** The size of the phase shift measured in milliseconds plotted against wavelength. Dark to light blue corresponds to the amplitude of the derivative curve, as in **(B–D)**. A horizontal line indicates that phase shift is independent of wavelength, i.e., fixed measured in milliseconds. For either all (dark blue) or most (light blue) of the λ range, data approach a flat line. For comparison, the duration of the mouse step cycle is 200–300 ms. **(F)** The size of the phase shift as a percentage of wavelength. To maintain a constant absolute phase shift, the size of the percentage shift varies with wavelength.

At any given instant in time, the rate and direction of change are given by the derivative of the Purkinje cell rate, i.e., the slope of a tangent to the rate curve at that time, derived by differentiation. The net effect of short-term plasticity accordingly has the same sign and varies (we assume linearly) with the derivative. Therefore, for example, it is momentarily nil when the direction of change switches, because the tangent at that time is horizontal. With a sinusoidal rate curve, it is nil twice a cycle, at the minimum and maximum Purkinje cell firing rates. The derivative of a sine function is another sine wave with the same frequency, 90 degrees (i.e., a quarter cycle) out of phase with the first, shifted to the left (Figure 6A).

It is not known whether the influence of rate dynamics fully displaces control by absolute rates in the behaving animal, or (more likely) net inhibition reflects the balance of their influence. The balance—net inhibition—is a function of the sum of the absolute rate and its derivative (itself representing net short-term plastic change), which we take as the sum itself to illustrate the effect. When sine waves with the same frequency are linearly combined, the result is a sine wave with the same wavelength which peaks somewhere in between (Figures 6B–D). The amount of the phase shift depends on their relative amplitude. The smaller the relative amplitude of the derivative curve (representing a modest effect of short-term plasticity), the smaller the shift from the rate curve, and vice versa. We illustrate the effect of adjusting relative amplitude of the derivative curve in Figures 6B–D.

We contend that by this mechanism, short-term plasticity causes DCN firing rates—cerebellar output—to be phase shifted to the left during locomotion. The result is to compensate for delay caused by extracerebellar transmission times, so that motor output of the cerebellum is received in phase with movement, without lag.

Because transmission time is short compared to the duration of a locomotion cycle, a modest phase shift may be sufficient to compensate for transmission times. Accordingly, the primary influence on DCN firing is provided by the absolute Purkinje cell rate, with an adjustment to reflect rate dynamics, we submit.

The duration of a locomotive cycle—wavelength—varies inversely in real time with the speed of the relative motion of body parts. There is an automatic real-time adjustment of phase shift for wavelength. Measured in degrees, shortening wavelength increases the size of the phase shift, and vice versa. Measured in milliseconds, however, the amount of the shift is virtually fixed (for wavelengths longer than ∼200 ms: Figure 6E). That is, the variation measured in degrees causes the shift in milliseconds to be independent of wavelength, as expected, because transmission times are constant.

## 7 Discussion

**FIGURE 7:**
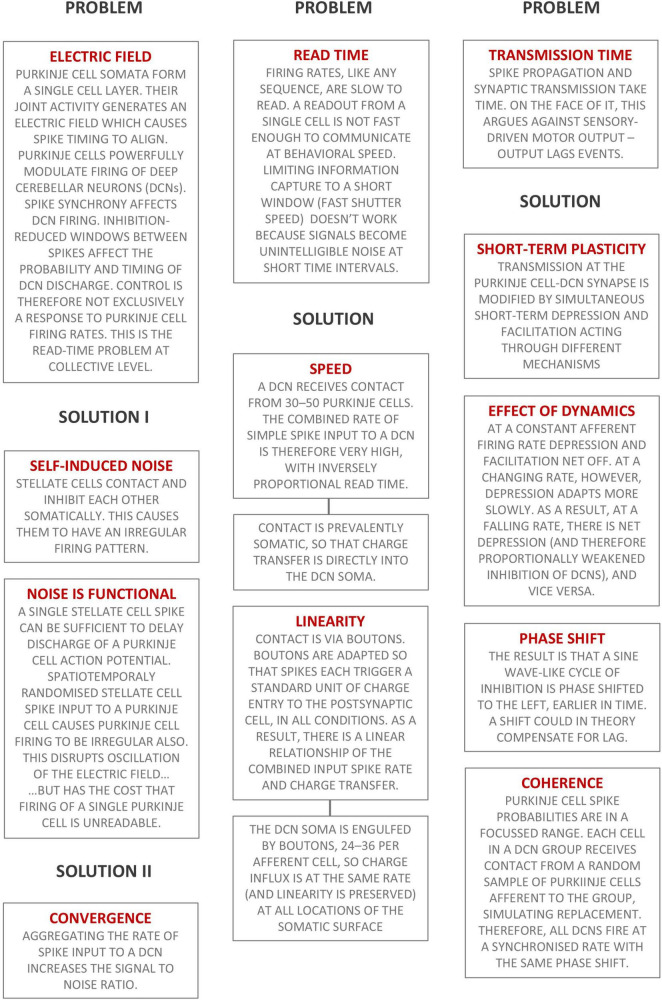
Summary of the main ideas presented in this paper. Text boxes headed ‘Problem’ state briefly a physiological design problem. Text boxes headed ‘Solution’ are the cerebellar solution.

Figure 7 is a schematic using text boxes to summarize the main ideas put forward in this paper.

### 7.1 The deep nuclei solve timing problems

“The naive concept of the cerebellar nuclei as a “simple” relay station that (inversely) forward signals computed in the cerebellar cortex…is now increasingly seen as lacking in depth” (Kebschull et al., 2024). We propose a functional view of the anatomy of communication between Purkinje cells and a deep nuclear cell group. This is the result of an attempt to explain the evidence and can be regarded as addressing two problems. First, coding information in neuronal firing rates (or any sequence) is inherently slow to read, too slow for behavioral speeds. Related to this, at short time intervals a readout becomes unreadable noise. How does the cerebellum decode inputs faster than it can read single signals? Second, signal transmission times cause delay, so motor commands lag events. How does the cerebellum compensate for signal transmission times so that motor outputs are timely?

We propose that the solution to slow read times is a passive computational effect of anatomy. Stated briefly, the combined input spike rate to a DCN is the sum of spike input from a random sample of Purkinje cells afferent to a nuclear group, so that DCNs individually receive an extremely high rate which is (i) therefore virtually instantaneously readable; and (ii) linearly related to the mean of afferent Purkinje cell rates; and so also (iii) synchronized in an output cell group.

The solution to transmission times, we argue, is short-term plasticity at the Purkinje cell-DCN synapse, which we explain as a mechanism which becomes active at a changing Purkinje cell firing rate, when the net effect depends on the direction and speed that the rate is changing. In a locomotor cycle, the result is to phase shift the cycle of inhibition of a DCN group to the left, compensating for lag.

### 7.2 Regarding compensation for transmission times

Compensation for transmission times depends on a dynamic Purkinje cell spike “rate.” Cyclic patterns, some of which are sinusoidal (some have more than one peak per cycle), have been reported in walking cats and rats (Edgley and Lidierth, 1988; Sauerbrei et al., 2015). Cerebellospinal neurons provide “a direct link between the cerebellum and spinal substrates for motor coordination” (Sathyamurthy et al., 2020). The output pathway is reciprocated by spinocerebellar neurons, providing a substrate for direct motor control. Neurons of the spinocerebellar tracts are excited monosynaptically by group Ia and group Ib afferents that originate in muscle spindles and tendon organs, respectively (Sengul and Watson, 2015). Afferent nerves that innervate muscles signal muscle length and the rate of change of muscle length, so nerve endings generate a cyclic rising and falling firing rate which is received as input to the cerebellum.

Lag compensation does not discriminate between inputs to a network, suggesting that travel times of inputs are not significantly variable or mixed. Signals originating in afferent nerves that excite the spinocerebellar tract, which enter the spinal cord in the same segment or neighboring segments, travel the same distance by the same pathway. Fast transmission reduces the effect of variable distance on timing. Internally generated information about movement is relayed by “large diameter, myelinated, afferent nerve fibers innervating specialized mechanoreceptors called muscle spindles and Golgi tendon organs” (Loeb and Mileusnic, 2015).

In the opposite direction, descending outputs that innervate a single motor neuron pool meet the same criteria. The number of output cells of a single network is modest. Descending motor signals are received by motor neuron pools, internally undivided columnar spinal nuclei, which convert excitation into proportionate recruitment and contraction of muscle fibers. In a straightforward system, this would provide the substrate for a cerebellar circuit to have a one-to-one relationship with a muscle, since a motor neuron pool innervates a single muscle, and a muscle receives innervation by a single motor neuron pool. In a more sophisticated system and perhaps later development, a motor neuron pool may receive input from more than one DCN group, but a DCN group has output to only one motor neuron pool.

We make these observations to suggest a possible topography that would allow motor circuits to incorporate compensation for transmission times. We submit that the deep cerebellar nuclei provide the hardware for precise timing associated with motor coordination.

### 7.3 Regarding other models

Our modeling envisages that microzone-grouped Purkinje cell spike probability is narrowly focussed, as suggested by a model of the cerebellar granular layer computation (Gilbert and Rasmussen, 2025, in press). In that model, granule cell encoded information is coded in the joint activity of large cell groups in the shape of long strips which mirror microzonal organization. The result is that the Purkinje cell population of a microzone downstream all receive, at any time, the same parallel fiber code. Control of Purkinje cells is rate coded.

On the face of it, this is in conflict with supervised learning models which have had a strong influence on thinking about the cerebellum, and which some researchers regard as an accepted understanding, and also with assumptions often made by artificial neural network models. In those models, learning under externally sourced instruction signals (hence “supervised”) teaches synaptic changes of transmission strength. Following training, the naive response to “remembered” patterns (inputs to training modified synapses) is displaced by a learned response. The function is to actively supplant control by firing rates. There is no proposal that microzone-grouped Purkinje cells receive the same code, or mechanism for them to fire at coherent rates. The ideas presented here are probably therefore not readily reconciled with those models.

### 7.4 A note on DCN recurrent collaterals

It feels counterintuitive that a cell group would have monosynaptic input to itself via axon collaterals. It would seem to achieve only that they receive information they already have. The function is to synchronize firing rates in a group. In this view, recurrents that angle back to make dendritic contact on other cells in the group provide background excitatory tone which reflects an average of firing rates of a random sample of other cells. The effect is to further tighten the focus of DCN rates by adding a second “layer” of averaging. In the first, DCNs randomly sample Purkinje cells afferent to the group, and in the second they randomly sample their neighbors, averaging the outputs of the first step. The precision this seeks to achieve is related to the contribution of the cerebellum to fine control and smooth execution of movements.

## Data Availability

The datasets presented in this study can be found in online repositories. The names of the repository/repositories and accession number(s) can be found below: doi: https://doi.org/10.25500/edata.bham.00001167 University of Birmingham Institutional Research Archive.
